# Use of multiple pharmacodynamic measures to deconstruct the Nix-TB regimen in a short-course murine model of tuberculosis

**DOI:** 10.1128/aac.01010-23

**Published:** 2024-03-19

**Authors:** M. A. Lyons, A. Obregon-Henao, M. E. Ramey, A. A. Bauman, S. Pauly, K. Rossmassler, J. Reid, B. Karger, N. D. Walter, G. T. Robertson

**Affiliations:** 1 Department of Microbiology, Immunology and Pathology, Mycobacteria Research Laboratories, Colorado State University, Fort Collins, Colorado, USA; 2 Division of Pulmonary Sciences and Critical Care Medicine, University of Colorado Anschutz Medical Campus, Aurora, Colorado, USA; 3 Consortium for Applied Microbial Metrics, Aurora, Colorado, USA; 4 Rocky Mountain Regional VA Medical Center, Aurora, Colorado, USA; Bill & Melinda Gates Medical Research Institute, Cambridge, Massachusetts, USA

**Keywords:** tuberculosis, pharmacodynamics, pretomanid, bedaquiline, linezolid, mouse model, RS ratio, interaction

## Abstract

A major challenge for tuberculosis (TB) drug development is to prioritize promising combination regimens from a large and growing number of possibilities. This includes demonstrating individual drug contributions to the activity of higher-order combinations. A BALB/c mouse TB infection model was used to evaluate the contributions of each drug and pairwise combination in the clinically relevant Nix-TB regimen [bedaquiline-pretomanid-linezolid (BPaL)] during the first 3 weeks of treatment at human equivalent doses. The rRNA synthesis (RS) ratio, an exploratory pharmacodynamic (PD) marker of ongoing *Mycobacterium tuberculosis* rRNA synthesis, together with solid culture CFU counts and liquid culture time to positivity (TTP) were used as PD markers of treatment response in lung tissue; and their time-course profiles were mathematically modeled using rate equations with pharmacologically interpretable parameters. Antimicrobial interactions were quantified using Bliss independence and Isserlis formulas. Subadditive (or antagonistic) and additive effects on bacillary load, assessed by CFU and TTP, were found for bedaquiline-pretomanid and linezolid-containing pairs, respectively. In contrast, subadditive and additive effects on rRNA synthesis were found for pretomanid-linezolid and bedaquiline-containing pairs, respectively. Additionally, accurate predictions of the response to BPaL for all three PD markers were made using only the single-drug and pairwise effects together with an assumption of negligible three-way drug interactions. The results represent an experimental and PD modeling approach aimed at reducing combinatorial complexity and improving the cost-effectiveness of *in vivo* systems for preclinical TB regimen development.

## INTRODUCTION

Pulmonary tuberculosis (TB) is treated using combinations of three or more antimicrobial drugs (higher-order drug combinations) taken daily or intermittently for at least 4–6 months (1, 2). There is a need, however, for shorter and more effective treatment regimens, especially for drug-resistant disease (3, 4). Mouse TB infection models are the primary *in vivo* systems for assessing the efficacy of new TB drugs and regimens (5, 6). They generate experimental data on pathophysiology, pharmacokinetics (PK), pharmacodynamics (PD), and relapse; and can guide allometric scaling and dose selection for early-phase clinical trials (7–11). Bactericidal activity of one or more drugs during early treatment is assessed by equilibrium exposure-response relationships, or by response-time profiles with sampling throughout the study duration (12, 13). The sterilizing or treatment-shortening activity of regimens is typically expressed as time required to prevent relapse in 95% of mice in the relapsing mouse model which treats mice for varying durations and then quantifies microbiologic relapse after a drug holiday (12). These study types provide a basis for prioritizing promising regimens and demonstrating individual drug contributions to the activity of a combination; with the latter being critical to the development of regimens that may contain multiple new investigational drugs (14).

While there are several established and novel methods for murine model evaluation of TB drugs and regimens (15, 16), their use for an exhaustive comparison between the hundreds of currently possible (17) higher-order combinations would be prohibitively expensive and time-consuming. Additionally, individual drug contributions to a combination are conventionally assessed by a process of single-drug additions and replacement (18, 19), which only partially accounts for antimicrobial interactions. Quantitative methods that assess the effects of higher-order combinations based on their single-drug and pairwise components have been described for several types of antibiotics using *in vitro* systems (20, 21). While these methods can be viewed as extensions of Bliss independence and Loewe additivity (as reference or null models for combined action of single drugs) (22), they have been applied in a predictive manner to reduce factorial measurements in prioritizing higher-order drug combinations (23, 24). Their extension to *in vivo* time-kill systems could simplify combinatorial complexity in regimen-based development, and better account for the combined action of the individual drugs within a combination.

The present study describes a quantitative evaluation of early treatment response to a higher-order anti-TB combination regimen based on single-drug and pairwise combination effects in a murine TB model. A BALB/c mouse high-dose aerosol subacute TB infection model was used to examine the approach with the possible combinations of bedaquiline (B), pretomanid (Pa), and linezolid (L) during the first 21 days of treatment. PD response in lung tissue was assessed by solid culture CFU counts, liquid culture time to positivity (TTP), and the RS ratio—an exploratory marker that indicates the rate of bacterial rRNA synthesis; with the ratio being the amount of unstable precursor rRNA (pre-rRNA) spacer sequence to stable mature rRNA (25). While changes in CFU and TTP are commonly used to assess bactericidal activity, the change in RS ratio during the first weeks of treatment has been shown to correlate with treatment-shortening activity in BALB/c mouse experiments (16, 25). The results included mathematical modeling of the time-course profiles for each PD marker, which provided for comparisons between individual drug contributions to the activity of the bedaquiline-pretomanid-linezolid (BPaL) combination; and antimicrobial interactions quantified relative to Bliss independence for each drug pair and an Isserlis formula for the higher-order combination (20). As the BPaL (Nix-TB) regimen was recently approved for the treatment of highly drug-resistant TB (26, 27), the results provide a clinically relevant starting point for an expanding library of single-drug and pairwise measurements from which predictions of novel higher-order combination regimens can be made and prioritized for further nonclinical or early-phase clinical testing.

## RESULTS

### Observed data: PD response-time profiles in BALB/c mice

**Fig 1:**
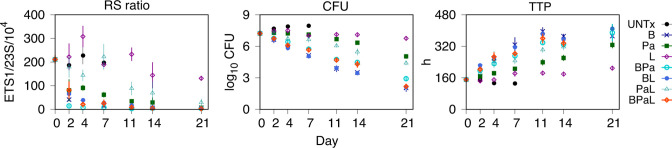
Combined treatment group response profiles of *M. tuberculosis* RS ratio, solid culture CFU, and liquid culture TTP from BALB/c mouse lungs. Data are the group mean (points) and SD (error bars) of the observed PD response versus treatment day. UNTx, untreated; B, bedaquiline; Pa, pretomanid; L, linezolid; BPa, bedaquiline-pretomanid; BL, bedaquiline-linezolid; PaL, pretomanid-linezolid; and BPaL, bedaquiline-pretomanid-linezolid.

The time course of PD response for each drug (B, Pa, L), drug pair (BPa, BL, PaL), and the three-drug BPaL combination was assessed by RS ratio, CFU, and TTP, from terminal sampling of lung tissue collected on treatment days 0 (baseline), 2, 4, 7, 11, 14, and 21; and for an untreated (UNTx) control group, on days 2, 4, and 7. Scatter plots of group mean and standard deviation (SD) values for each PD marker versus time are shown in Fig. 1. The general pattern of activity for log_10_CFU and TTP showed an inverse relationship, with monophasic decreases in the former matching corresponding increases in the latter; and which varied uniformly across the treatment groups. The RS ratio profiles showed a biphasic response with an initial rapid reduction that slowed toward a plateau by the end of the first week. For PaL, however, there was a slower rate of reduction without a clear inflection point; and for linezolid monotherapy, there was a pronounced increase from baseline to day 4 followed by a variable but downward trend.

### PD modeling: characterization of drug activity

**Fig 2:**
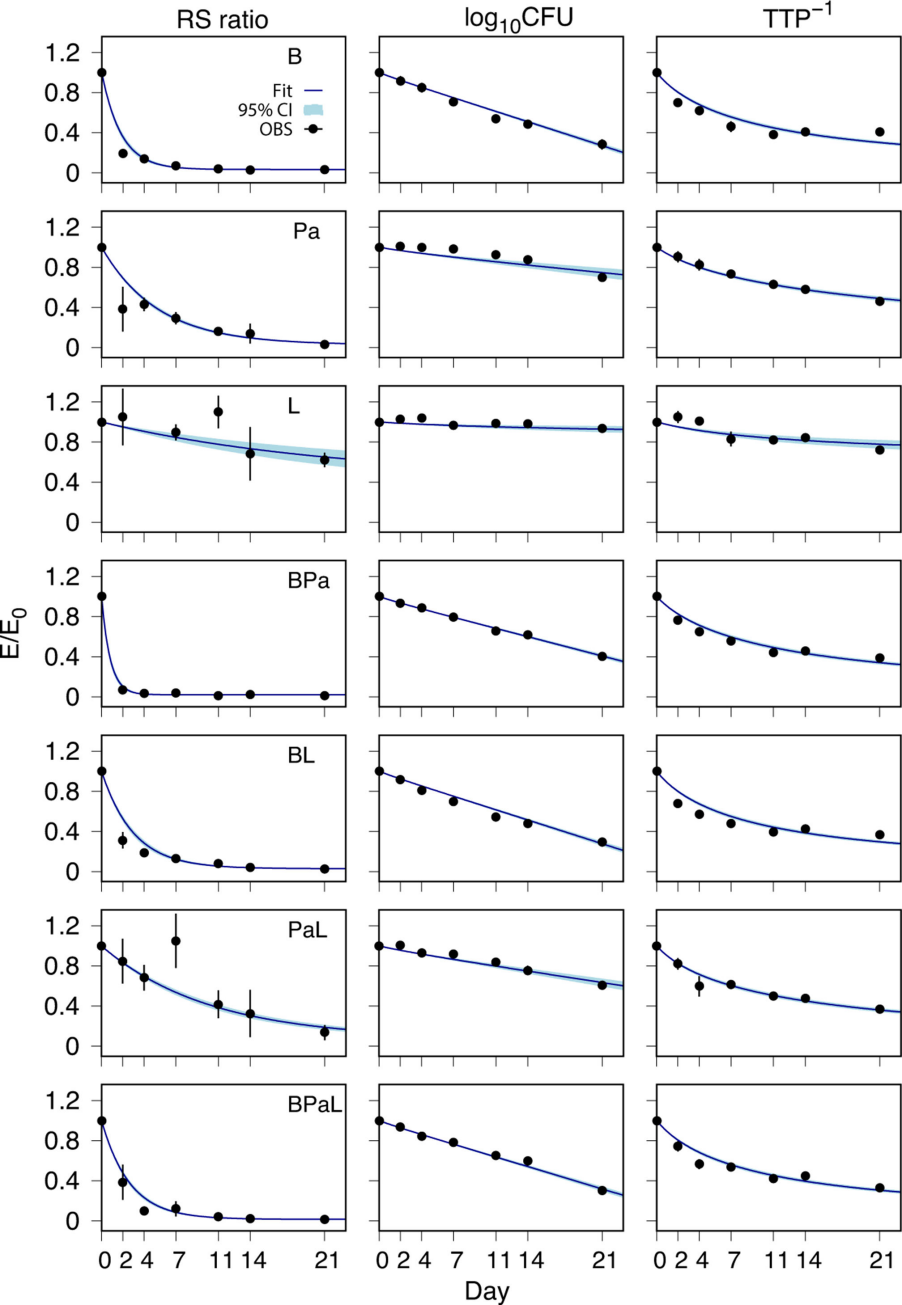
Fractional effect (*E*/*E*_0_) profiles of *M. tuberculosis* RS ratio, solid culture CFU (log_10_CFU), and reciprocal of the liquid culture TTP^−1^ from BALB/c mouse lungs. Data are model simulations of the predicted mean (solid line with shaded 95% confidence interval) together with observed group mean (points) and SD (error bars) for each treatment group versus time. The observed day 4 RS ratio mean (SD) value for L not shown in the plot was equal to 1.46 (0.25). B, bedaquiline; Pa, pretomanid; L, linezolid; BPa, bedaquiline-pretomanid; BL, bedaquiline-linezolid; PaL, pretomanid-linezolid; and BPaL, bedaquiline-pretomanid-linezolid.

The PD response profiles were parameterized using rate equations based on synthesis and degradation of pre-rRNA spacer sequences assessed by RS ratio, and bacterial population growth and drug-killing effect assessed by CFU and TTP. The model parameters were estimated as probability distributions conditioned on the baseline-normalized group mean values of each PD marker and are summarized by their distribution mean and SD in Table 1. The rate and extent of drug effect on RS ratio were characterized by a degradation half-life, $t_{1/2}^{RS}$, and an equilibration value, RS_ss_, respectively. The half-life characterizes the initial treatment effect and depends inversely on the degradation rate, while the equilibration value represents the long-term equilibrium state. Bactericidal activity was characterized by the drug kill rate constant, *k*_drug_, which represents the rate of log_10_CFU/lung decrease or TTP increase; together with a solid-liquid culture conversion factor, *τ*, where the liquid culture was a mycobacteria growth indicator tube (MGIT) system. For a particular liquid culture inoculum with matching solid culture CFU, smaller and larger values of *τ* represent, respectively, larger and smaller rates of oxygen consumption yielding corresponding smaller and larger TTP values. The mean (SD) values for the bacterial population growth rate constant, *µ* = 0.72 (0.043)/day, and carrying capacity, *K* = 9.34 × 10^7^ (5.83 × 10^6^) CFU/lung in the absence of drug were determined from the untreated group data (Fig. S1). Model simulations of the baseline-normalized PD profiles as fractional effects, *E*/*E*_0_, are shown together with the observed data in Fig. 2; where *E* = *E*(*t*) is the PD response at elapsed time, *t*, from the first dose, and *E*_0_ = *E*(*t* = 0) is the baseline value. The fractional effect for TTP was defined using the reciprocal values, TTP^−1^.

**TABLE 1:** PD model parameter values for bactericidal activity and RS ratio turnover for each treatment group^*a*^

	Parameter (units)			
Group	$k_{drug}$ (1/d)	*τ* (h)	$t_{1/2}^{RS}$ (d)	${RS}_{ss}$ (ETS1/23S/10^4^)
B	1.29 (0.02)	28.7 (1.2)	1.4 (0.1)	6.1 (1.0)
Pa	0.90 (0.02)	37.5 (3.5)	3.5 (0.3)	7.4 (1.7)
L	0.71 (0.03)	38.0 (7.7)	13 (2.4)	65 (23)
BPa	1.18 (0.01)	29.6 (1.3)	0.69 (0.06)	3.7 (0.6)
BL	1.28 (0.01)	29.8 (1.3)	2.1 (0.2)	6.0 (1.2)
PaL	0.99 (0.02)	44.0 (2.9)	6.5 (0.58)	18 (5)
BPaL	1.25 (0.01)	30.2 (1.3)	1.8 (0.1)	3.7 (0.7)

^*a*^ Data are posterior distribution mean (SD), sample size equal to 10,000. *k*_drug_, drug kill rate constant; *τ*, solid-liquid culture time constant; $t_{1/2}^{RS}$, RS ratio half-life; RS_ss_, RS ratio equilibration constant; B, bedaquiline; Pa, pretomanid; L, linezolid; BPa, bedaquiline-pretomanid; BL, bedaquiline-linezolid; PaL, pretomanid-linezolid; BPaL, bedaquiline-pretomanid-linezolid.

As single drugs, the effects of B, Pa, and L were distinguished by differences in their PD parameter values and by their profile shapes, with bedaquiline monotherapy showing the largest kill rate, and the most rapid and extensive reduction in RS ratio. While the kill rate constants and overall time course for decline in bacillary burden in the lungs were similar for B, BPa, BL, and BPaL, they show pronounced differences in their effect on RS ratio degradation rate and equilibration values with BPaL showing the smallest equilibration value. The most rapid effect on RS ratio was observed for mice treated with BPa, which was antagonistic by CFU and TTP measures relative to bedaquiline given as monotherapy. Overall, the RS ratio parameter values and time-course profiles exhibited more clearly defined differences between the various tested regimens that were less apparent based on CFU and TTP kill curves alone.

While the PD model assumptions provided an explanatory representation of basic processes and features of the observed profiles for each PD marker, the relatively small but visually apparent errors, or differences between model simulation and observed data, in the first week of treatment indicate transitory drug effect processes that were not accounted for in the model equations. While not especially large, the deviations between model output and observation indicate delayed onset in bactericidal activity in the first-week CFU data for Pa, L, and PaL. Similarly, differences between modeled and observed RS ratios for B, BL, and BPaL on day 2 and day 4 indicate a possible additional early-phase synthesis-degradation process for bedaquiline exposure. The largest deviation from the modeling assumptions is seen for the RS ratio response to linezolid monotherapy, with the first-week increase from baseline indicating a delayed onset of degradation. The errors in the first week of treatment for TTP indicate possible limits on the assumption of similarity between the viable bacilli in the two culture media; such differences in mycobacterial cell populations have been characterized for rifampicin in human early bactericidal activity (EBA) studies (28). After the first week of treatment, however, the modeling errors are smaller and less informative for drug effect processes that deviate from the modeling assumptions. This suggests the attainment of equilibrium in drug effects after the first week with response profiles accounted for by simple assumptions of first-order drug kill rates, equivalence between bactericidal activity assessed by log_10_CFU and TTP, and a balance between synthesis and degradation of precursor rRNA.

### PD interactions

**Fig 3:**
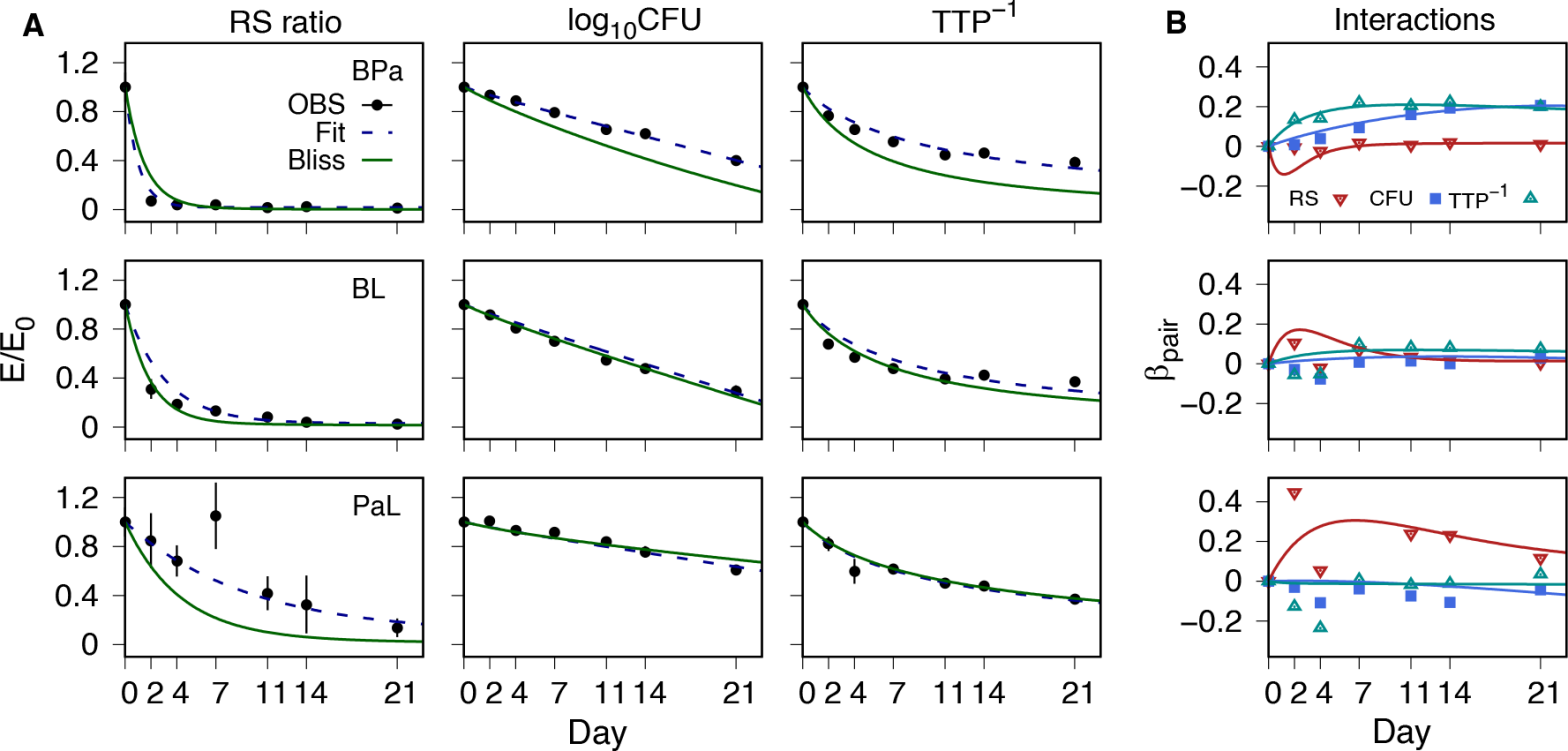
Pairwise PD response profiles and interactions as a deviation from Bliss independence. (A) PD marker fractional effect (*E*/*E*_0_) for bedaquiline-pretomanid (BPa), bedaquiline-linezolid (BL), and pretomanid-linezolid (PaL) combinations. Observed data (OBS) group mean (points) and SD (error bars), together with model simulations for the pair (dashed lines) and Bliss independence as pointwise products of single-drug model fits. (B) Interactions as deviations from Bliss independence. Values are shown for each PD marker for the observed mean values (point symbols) and model predictions (lines).

Time-course profiles of the PD interactions were determined using the fractional effect profiles for RS ratio, log_10_CFU, and TTP^−1^. A basic difference between the methods of Loewe and Bliss is the requirement of dose-response curves for the former, while the latter requires only single-dose measurements. As such, Bliss independence and its statistical interpretation could be applied directly to our single-dose kinetic profiles for the drug pairs. Figure 3 shows the observed and modeled PD marker data for each drug pair and the pointwise product of the modeled single-drug profiles, where the latter corresponds to an additive effect relative to Bliss independence (statistical independence). Also shown are the interactions calculated as the difference between the two PD response curves, or deviation from additivity, together with the corresponding observed data. Positive deviations represent subadditive (or antagonistic) interactions, resulting from a smaller effect observed for the drug pair than expected by the product of their single-drug profiles. Subadditive interactions were found for the BPa CFU and TTP profiles, and for PaL in the RS ratio. All other pairwise effects were found to be approximately additive, without substantive differences between the observed values and those predicted from an independent product assumption. For BPa, the interactions exhibited an initial increasing and variable response that became stable by day 11.

**Fig 4:**
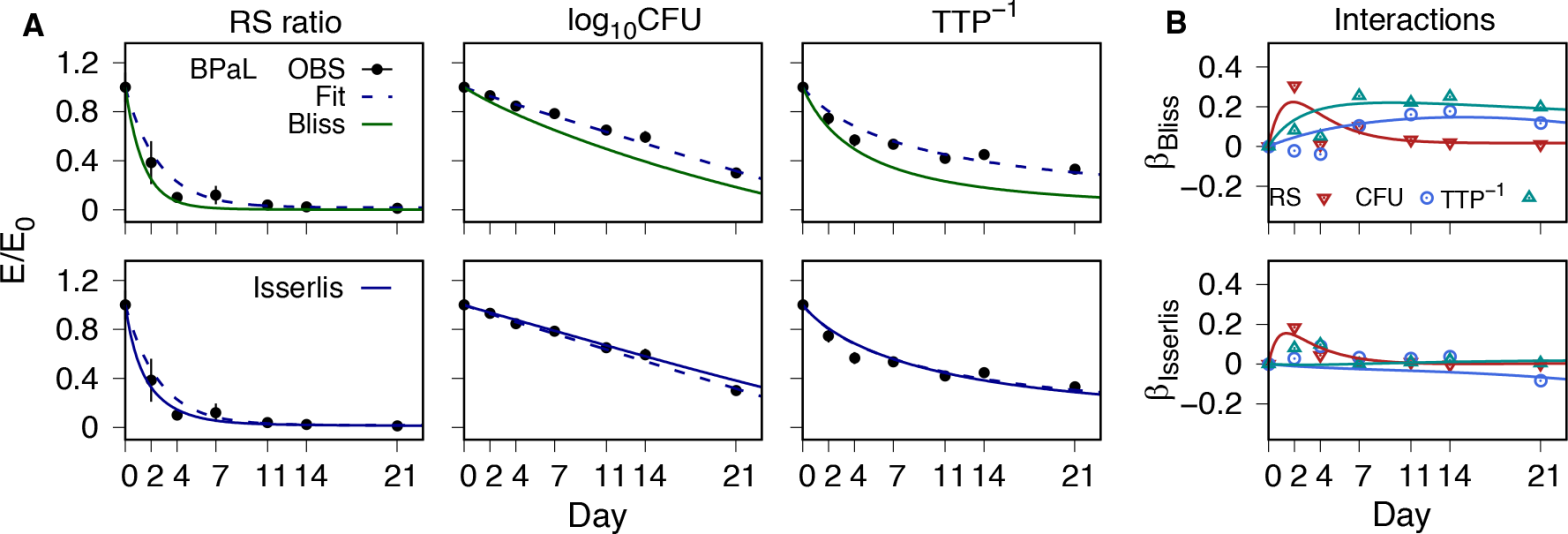
BPaL PD response profiles and interactions as deviations from Bliss and Isserlis formulas. (A) PD marker fractional effect (*E*/*E*_0_) for BPaL. Observed data (OBS) group mean (points) and SD (error bars), together with model simulations for the combination (dashed lines) and Bliss independence (top row) and Isserlis formula predictions (bottom row). (B) Interactions as deviations from Bliss independence (top row) and Isserlis formula (bottom row). Values are shown for each PD marker for the observed mean values (point symbols) and model predictions (lines).

The extension of Bliss independence to three drugs becomes the triple product of the single-drug effects, which does not account for mutual interactions. The Isserlis formula includes the measured pairwise effects without assumptions on the type or magnitude of their interactions. Figure 4 shows the observed BPaL data and PD model fits together with the model-predicted Isserlis and Bliss formulas. Relative to Bliss independence, the combination was found to be subadditive with respect to CFU and TTP, and except for the day 2 value, additive with respect to RS ratio. In contrast, and again with the exception of the day 2 RS ratio value, there were no significant three-way interactions for BPaL when the mutual interactions were accounted for by the Isserlis formula. The absence of three-way interactions used as an assumption (20) provided accurate predictions of BPaL response for all three PD markers based on the responses of the single-drug and pairwise components.

## DISCUSSION

While murine models provide a critical assessment of new TB drugs and treatment regimens leading up to clinical testing, they can be costly and labor-intensive, which limits their use as high-throughput screens for the large numbers of possible higher-order combination regimens generated from the current drug pipeline (29, 30). The purpose of this study was to examine the use of single-drug and pairwise combinations as a reduced set of targeted drug effect measurements to predict the outcomes of higher-order drug combinations and to demonstrate the component drug contributions to the PD responses of the regimen as a whole. A standard mouse TB infection model with three different PD markers, and the BPaL regimen, was used as a test case. The most common methods for predicting higher-order antibiotic combination effects are based on *in vitro* equilibrium studies measuring bacterial population growth under subinhibitory drug concentrations (20, 21, 24, 31). Here, these methods were adapted to an *in vivo* system with PD response-time profiles and bactericidal concentrations. The results included accurate predictions of BPaL effects on the kinetic profiles of the three PD markers, the characterization of BPaL component drug contributions to the combination based on a minimal set of pharmacologically interpretable parameters, and quantitative measures of drug interactions using two different models for additivity. These results show a potential for single-drug and pairwise measurements to predict higher-order combination effects as an experimentally feasible approach to prioritizing large numbers of novel TB regimens for further nonclinical and early-phase clinical testing; including combinations that would otherwise remain untested.

Demonstration of the individual drug contributions to the activity of a combination is a basic task for the development of novel TB regimens (14). For mouse TB models, such demonstration is typically conducted one drug at a time, where efficacy of the combination is assessed with and without each tested drug (18, 19, 32). Separate from investigation of its predictive capacity, the Isserlis formula provided a deconstruction of the PD effects of BPaL as a sum of effects arising from single-drug and pairwise products. The PD effects were also characterized for each drug component by a kill rate constant for bactericidal activity, and RS ratio half-life and equilibration as a measure of rRNA synthesis (16, 25). The interactions were quantified over time as deviations from Bliss and Isserlis formulas, with the latter including mutual interactions in contrast to the former which did not. Pretomanid was the only substantively interacting drug, with subadditive effects on bacillary load in combination with bedaquiline, and on rRNA synthesis in combination with linezolid. There were no substantive interactions among the three PD markers for the BL pair, nor any that emerged from all three drugs acting together when mutual interactions were accounted for. The different characterizations of BPaL as subadditive by Bliss independence and additive by the Isserlis formula illustrate the dependence of such classification on how combined action without interaction is defined. The difference in this case is illustrated by the predictive accuracy of the Isserlis formula compared to Bliss independence. Additionally, such interactions were found to differ depending on the type of PD response, which shows the utility of including multiple PD markers, in this case for rRNA synthesis using the RS ratio and burden using CFU and TTP.

Beyond the methodological limitations in data handling, modeling assumptions, and the sensitivity to variability and error, the main limitation to generalizing the results of this study is the narrow scope, with only one drug combination, one dosage regimen, and one *M. tuberculosis* strain; where strain differences, in particular, may result in different outcomes for the PD interactions as have been observed for BALB/c TB-infection models (33). Evaluation of a wider range of drug classes, including dose ranging for at least the single-drug measurements, would better establish a basic pattern of outcomes from this experimental system. While available mouse studies of BPaL have not included possible PK drug-drug interactions (18, 34), the absence of such PK measurements is a limitation on understanding sources of error in the observed PD interactions. Application of the Isserlis formula for prediction required an assumption of negligible higher-order interactions and was based on previous observation of such effects from *in vitro* antibiotic combination studies including antimycobacterial drugs (20, 21, 24), and from theoretical considerations of locality (35) that might apply to drug-mediated interactions in biochemical processes. While the accuracy of the BPaL results across disparate PD markers may indicate a more general application, there are examples of higher-order or emergent effects that challenge this assumption (31, 36). For example, factorial measurements of *in vitro* antibiotic growth inhibition of *Escherichia coli* identified emergent interactions in 17% of 1,512 three-drug combinations with the proportion increasing with the number of drugs (37). A comparison between growth inhibition and bactericidal activity of these combinations could improve understanding of the limitations on predictive capacity of pairwise interactions. Although the combination of CFU and RS ratio reduction has been shown to correlate with treatment-shortening ability for several different combination regimens (16, 25), the absence of selection criteria beyond that of maximum observed effect is another limitation. For example, possible relationships between PD interactions and acquired drug resistance may require a more detailed understanding of resistance mechanisms and longer treatment duration to assess (18, 38). Application of this analysis to other PD markers such as lipoarabinomannan (39) and 16S rRNA (40) remains untested.

This study combined *in vivo* measurements of drug effect with mathematical modeling as a potential method of prioritizing novel TB regimens for further nonclinical and clinical evaluation. The experimental design was similar to phase 2a EBA studies (41) and could be further combined with similarly designed *in vitro* time-kill studies to identify translatable elements across the preclinical and early-phase clinical development stages. Consideration of the planned evaluation of novel TB regimens by the UNITE4TB consortium (42) provides an example of how the approach developed here could be applied. There are eleven planned phase 2b/c studies of mostly four-drug combinations assembled from eight individual drugs assessed in phase 2a monotherapy testing. In total, 19 phase 2 studies will yield an evaluation of 11 combination regimens. Using Isserlis formulas based on single-drug and pairwise outcomes (20), an alternative approach could first measure the 8 single-drug and 28 pairwise combinations of the 8 drugs, which would then yield predictions for 56 three-drug, 70 four-drug, and 56 five-drug combinations. A ranking of promising regimens could be made from comparisons among predicted outcomes for early bactericidal activity using CFU or TTP and treatment-shortening using the combined CFU and RS ratio (16, 25), to provide a basis for additional nonclinical testing. Such an approach could provide a more thorough and systematic prioritization of combination regimens for early-phase clinical trials, and the flexibility to incorporate new drugs with minimal experimental testing.

## MATERIALS AND METHODS

### BALB/c mouse high-dose aerosol infection model

All animal studies were performed at Colorado State University in a certified animal biosafety level III facility, and conducted in accordance with guidelines of the Colorado State University Institutional Animal Care and Use Committee (reference number: 1212). Six- to eight-week-old female pathogen-free BALB/c mice (Jackson Laboratories) were exposed to high-dose aerosol (Glas-Col) of *M. tuberculosis* Erdman from broth culture (optical density at 600 nm of ∼0.8) to achieve deposition of ∼4.0 log_10_CFU in the lungs of each mouse (43). Mice were sacrificed for lung CFU counts 1 day after infection (day −10) and at day 0 to determine the number of CFU implanted in lungs and the number of bacilli present at the start of treatment, respectively.

### Antibiotic treatment

Mice were randomized to the different treatment groups. Treatments were initiated 11 days post-aerosol (day 0) and were given once daily by oral gavage, 7 days per week, for the duration of the study. Bedaquiline fumarate was administered at 25 mg/kg. Linezolid was administered at 100 mg/kg. Pretomanid was administered at 50 mg/kg. Bedaquiline fumarate was formulated in 20% hydroxypropyl-b-cyclodextrin solution acidified with 1.5% 1N HCl. Pretomanid was prepared in the CM-2 formulation as previously described (43). Linezolid was prepared in 5% polyethylene glycol (PEG)-200/95% (0.5%, wt:vol) methyl-cellulose. In cases of drug combinations, bedaquiline was administered first, followed 1 h later by pretomanid and/or 4 h later by linezolid. Each drug was administered in 0.2 mL volume.

### Evaluation of drug activity

Drug bactericidal activity was assessed by lung CFU reductions and TTP increases, and by RS ratio turnover. Groups of five mice each were humanely euthanized 1 day following 2, 4, 7, 11, 14, or 21 consecutive days of drug treatment. At each time point, lung lobes were removed aseptically. The superior and middle lobes (roughly one-third of the lung by mass) were immediately flash frozen under liquid nitrogen for RNA preservation. The remaining two-thirds of the lung were used for CFU and TTP assessments. Details on RS ratio assessment were described previously (25). For CFU plating and TTP assessments, previously frozen lungs were homogenized using the Bertin Precellys CKMix50-7 mL lysis kit [in 4.5 mL phosphate-buffered saline (PBS) with 10% bovine serum albumin (BSA)]. Lung homogenates were plated in serial dilutions on 0.4% charcoal-supplemented 7H11 agar supplemented with 10% oleic acid, BSA, dextrose, and catalase (OADC) and with selective antibiotics, including cycloheximide (10 mg/L) and carbenicillin (50 mg/L).

For TTP assessments, lung homogenates were diluted fivefold in 7H9 media supplemented with 10% OADC and 0.05% Tween80, placed in a 2 mL screw-cap tube containing sterile 2 mm beads and bead-beat at 2,800 rpm for 10 s. Homogenates were then inoculated into 7 mL MGITs containing 7H9 media supplemented with 10% OADC, 0.05% Tween80, 0.4% charcoal, and BD MGIT PANTA antibiotic mixture (polymyxin B, amphotericin B, nalidixic acid, trimethoprim and azlocillin), per the manufacturer’s instructions. Finally, MGITs were incubated in the BD BACTEC MGIT 320 system for at least 42 days at 37°C. TTP was recorded as the time (in h) for cultures to turn positive. Cultures that were assayed in the MGIT system but remained negative up to day 42 (the accepted cutoff) were recorded as negative.

### PD modeling and antimicrobial interactions

The PD response profiles for each treatment group were modeled as functions of elapsed time, *t*, from the start of treatment using PD rate equations (44, 45). The PD response for RS ratio was represented by a turnover equation:


$$\frac{dR}{dt}=k_{prod}-k_{loss}\cdot R$$

where *R* = *R*(*t*) is the RS ratio with initial condition, *R*(0) = *R*_0_. The rate constants for apparent production, $k_{prod}$, and fractional loss, $k_{loss}$, were expressed as a half-life, $t_{1/2}^{RS}=\ln\;(2)/k_{loss}$ , and an equilibration (or steady state) value, ${RS}_{ss}=k_{prod}/k_{loss}$. The CFU and TTP profiles were modeled using logistic growth with constant drug-killing effect as follows:


$$\frac{dN}{dt}=\mu \cdot \left(1-\frac{N}{K}\right)\cdot N-k_{drug}\cdot N$$

$$\frac{dT}{dt}=-\tau \cdot \left[\mu \left(1-\frac{N_{0}}{K}exp[-(T-T_{0})/\tau ]\right)-k_{drug}\right]$$

where *N* = *N*(*t*) represents the CFU/lung and *T* = *T*(*t*) is the TTP. The initial conditions were specified as *N*(0) =*N*_0_ and *T*(0) =*T*_0_. The parameter, *µ*, is the bacterial growth rate constant; *K*, the carrying capacity; $k_{drug}$, the drug kill rate constant; and *τ*, a solid-liquid culture conversion factor between CFU and TTP with a detailed derivation previously described (46). The model parameters were estimated from the observed data using Bayes’ theorem and Markov chain Monte Carlo (MCMC) simulation (47). The MCMC simulations consisted of 10 independent sampling chains of 100,000 iterations each; with every 10th iteration of the last 10,000 retained and aggregated into a final 10,000 iteration sample.

The PD interactions were characterized as functions of time using the fractional response (or survival fraction), *u* = *u*(*t*), for each drug alone, *u*_*i*_, the pairs, *u*_*ij*_, and the three-drug combination, *u_ijk_*, where *i*, *j*, *k* are drug labels. Pairwise interactions were quantified as the deviation from Bliss independence, *β_ij_* = *u_ij_* − *u_i_u_j_*, and three-way interactions as deviations from an Isserlis formula (20), *β_ijk_* = *u_ijk_* − (*u_i_u_jk_* + *u_j_u_ki_* + *u_k_u_ij_* − 2*u_i_u_j_u_k_*).

In the absence of pairwise interactions, the Isserlis formula reduces to a Bliss independence formula with deviations *β_ijk_* = *u_ijk_* − *u_i_u_j_u_k_*. Interactions were specified relative to zero deviation (additive) as subadditive (or antagonistic) for positive deviation or superadditive (or synergistic) for negative deviation.

### Data handling and limitations

The observed mouse efficacy data were obtained as terminal samples, which precluded individual PD response-time profiles. The PD profiles used for model parameter estimates were constructed as population summaries from the group mean values calculated with missing data ignored. Negative cultures were treated as missing. The mathematical models were constructed as simple representations of the basic biological processes underlying the observed PD responses during early treatment. Additional features or distinguishing factors related to drug mechanisms of action, such as delays in onset of activity, multiple subpopulations of bacilli, or features that may become evident with longer-duration treatments such as a possibly vanishing RS ratio, were not included in the PD modeling assumptions. The relationship between CFU and TTP included an assumption of similarity between the viable bacillary populations that are detected in the two different culture media, and that this relationship did not change over time. The Isserlis and Bliss formulas are sensitive to measurement uncertainty and variability due to multiplication of error (48).

### Software

Model simulations and data analysis were implemented using MCSim Modeling and Simulation Suite (47) (version 6.2.0; http://www.gnu.org/software/mcsim), the R statistical software (version 3.3.3; R Development Team, https://www.R-project.org) with the CODA package (version 0.18; https://cran.r-project.org/web/packages/coda), and gnuplot (version 5.0; http://www.gnuplot.info/). The operating system was Linux (version 3.16.0-4-amd64; Debian distribution, https://www.debian.org).

## Data Availability

The individual mouse CFU, TTP, and RS ratio data and computer model simulation files are provided in the supplemental material.

## SUPPLEMENTAL MATERIAL

Data File S1 aac.01010-23-s0001.txt MCSim model definition file.

Data File S2 aac.01010-23-s0002.txt MCSim MCMC simulation file.

Data File S3 aac.01010-23-s0003.txt MCSim model simulation file.

Data File S4 aac.01010-23-s0004.txt Observed mouse CFU data.

Data File S5 aac.01010-23-s0005.txt Observed mouse RS ratio data.

Data File S6 aac.01010-23-s0006.txt Observed mouse TTP data.

Supplemental material aac.01010-23-s0007.pdf Untreated group data and model parameters.

ASM does not own the copyrights to Supplemental Material that may be linked to, or accessed through, an article. The authors have granted ASM a non-exclusive, world-wide license to publish the Supplemental Material files. Please contact the corresponding author directly for reuse.

## References

[1] Nahid P, Dorman SE, Alipanah N, Barry PM, Brozek JL, Cattamanchi A, Chaisson LH, Chaisson RE, Daley CL, Grzemska M, et al.. 2016. Official American Thoracic Society/Centers for Disease Control and Prevention/Infectious Diseases Society of America Clinical Practice Guidelines: treatment of drug-susceptible tuberculosis. Clin Infect Dis 63:e147–e195. doi:10.1093/cid/ciw376 27516382 PMC6590850

[2] Dorman SE, Nahid P, Kurbatova EV, Phillips PPJ, Bryant K, Dooley KE, Engle M, Goldberg SV, Phan HTT, Hakim J, et al.. 2021. Four-month rifapentine regimens with or without moxifloxacin for tuberculosis. N Engl J Med 384:1705–1718. doi:10.1056/NEJMoa2033400 33951360 PMC8282329

[3] Ginsberg A. 2011. The TB Alliance: overcoming challenges to chart the future course of TB drug development. Future Med Chem 3:1247–1252. doi:10.4155/fmc.11.82 21859299

[4] Lienhardt C, Nahid P, Rich ML, Bansbach C, Kendall EA, Churchyard G, González-Angulo L, D’Ambrosio L, Migliori GB, Raviglione M. 2017. Target regimen profiles for treatment of tuberculosis: a WHO document. Eur Respir J 49:1602352. doi:10.1183/13993003.02352-2016 28122858 PMC5509341

[5] Gumbo T, Lenaerts AJ, Hanna D, Romero K, Nuermberger E. 2015. Nonclinical models for antituberculosis drug development: a landscape analysis. J Infect Dis 211:S83–S95. doi:10.1093/infdis/jiv183 26009617

[6] Nuermberger EL. 2017. Preclinical efficacy testing of new drug candidates, p 269–293. In Tuberculosis and the tubercle bacillus10.1128/microbiolspec.tbtb2-0034-2017 PMC11687513 28643624

[7] Lenaerts A, Barry CE, Dartois V. 2015. Heterogeneity in tuberculosis pathology, microenvironments and therapeutic responses. Immunol Rev 264:288–307. doi:10.1111/imr.12252 25703567 PMC4368385

[8] Yang H-J, Wang D, Wen X, Weiner DM, Via LE. 2021. One size fits all? Not in in vivo modeling of tuberculosis chemotherapeutics. Front Cell Infect Microbiol 11:613149. doi:10.3389/fcimb.2021.613149 33796474 PMC8008060

[9] Robertson GT, Ramey ME, Massoudi LM, Carter CL, Zimmerman M, Kaya F, Graham BG, Gruppo V, Hastings C, Woolhiser LK, Scott DWL, Asay BC, Eshun-Wilson F, Maidj E, Podell BK, Vásquez JJ, Lyons MA, Dartois V, Lenaerts AJ. 2021. Comparative analysis of pharmacodynamics in the C3HeB/Fej mouse tuberculosis model for DprE1 inhibitors TBA-7371, PBTZ169, and OPC-167832. Antimicrob Agents Chemother 65:e0058321. doi:10.1128/AAC.00583-21 34370580 PMC8522729

[10] Bartelink IH, Zhang N, Keizer RJ, Strydom N, Converse PJ, Dooley KE, Nuermberger EL, Savic RM. 2017. New paradigm for translational modeling to predict long-term tuberculosis treatment response. Clin Transl Sci 10:366–379. doi:10.1111/cts.12472 28561946 PMC5593171

[11] Ernest JP, Strydom N, Wang Q, Zhang N, Nuermberger E, Dartois V, Savic RM. 2021. Development of new tuberculosis drugs: translation to regimen composition for drug-sensitive and multidrug-resistant tuberculosis. Annu Rev Pharmacol Toxicol 61:495–516. doi:10.1146/annurev-pharmtox-030920-011143 32806997 PMC7790895

[12] Li SY, Tasneen R, Tyagi S, Soni H, Converse PJ, Mdluli K, Nuermberger EL. 2017. Bactericidal and sterilizing activity of a novel regimen with bedaquiline, pretomanid, moxifloxacin, and pyrazinamide in a murine model of tuberculosis. Antimicrob Agents Chemother 61:e00913-17. doi:10.1128/AAC.00913-17 28630203 PMC5571308

[13] Grosset J, Almeida D, Converse PJ, Tyagi S, Li S-Y, Ammerman NC, Pym AS, Wallengren K, Hafner R, Lalloo U, Swindells S, Bishai WR. 2012. Modeling early bactericidal activity in murine tuberculosis provides insights into the activity of isoniazid and pyrazinamide. Proc Natl Acad Sci U S A 109:15001–15005. doi:10.1073/pnas.1203636109 22927424 PMC3443175

[14] U.S. Food and Drug Administration Center for Drug Evaluation and Research. 2013. Guidance for industry: codevelopment of two or more new investigational drugs for use in combination

[15] Franzblau SG, DeGroote MA, Cho SH, Andries K, Nuermberger E, Orme IM, Mdluli K, Angulo-Barturen I, Dick T, Dartois V, Lenaerts AJ. 2012. Comprehensive analysis of methods used for the evaluation of compounds against Mycobacterium tuberculosis. Tuberculosis (Edinb) 92:453–488. doi:10.1016/j.tube.2012.07.003 22940006

[16] Dide-Agossou C, Bauman AA, Ramey ME, Rossmassler K, Al Mubarak R, Pauly S, Voskuil MI, Garcia-Cremades M, Savic RM, Nahid P, Moore CM, Tasneen R, Nuermberger EL, Robertson GT, Walter ND. 2022. Combination of Mycobacterium tuberculosis RS ratio and CFU improves the ability of murine efficacy experiments to distinguish between drug treatments. Antimicrob Agents Chemother 66:e0231021. doi:10.1128/aac.02310-21 35311519 PMC9017352

[17] Black TA, Buchwald UK. 2021. The pipeline of new molecules and regimens against drug-resistant tuberculosis. J Clin Tuberc Other Mycobact Dis 25:100285. doi:10.1016/j.jctube.2021.100285 34816020 PMC8593651

[18] Xu J, Li S-Y, Almeida DV, Tasneen R, Barnes-Boyle K, Converse PJ, Upton AM, Mdluli K, Fotouhi N, Nuermberger EL. 2019. Contribution of pretomanid to novel regimens containing bedaquiline with either linezolid or moxifloxacin and pyrazinamide in murine models of tuberculosis. Antimicrob Agents Chemother 63:e00021-19. doi:10.1128/AAC.00021-19 30833432 PMC6496099

[19] Li S-Y, Converse PJ, Betoudji F, Lee J, Mdluli K, Upton A, Fotouhi N, Nuermberger EL. 2023. Next-generation diarylquinolines improve sterilizing activity of regimens with pretomanid and the novel oxazolidinone TBI-223 in a mouse tuberculosis model. Antimicrobial Agents and Chemotherapy 67:e0003523. doi:10.1128/aac.00035-23 36920217 PMC10112056

[20] Wood K, Nishida S, Sontag ED, Cluzel P. 2012. Mechanism-independent method for predicting response to multidrug combinations in bacteria. Proc Natl Acad Sci U S A 109:12254–12259. doi:10.1073/pnas.1201281109 22773816 PMC3409729

[21] Zimmer A, Katzir I, Dekel E, Mayo AE, Alon U. 2016. Prediction of multidimensional drug dose responses based on measurements of drug pairs. Proc Natl Acad Sci U S A 113:10442–10447. doi:10.1073/pnas.1606301113 27562164 PMC5027409

[22] Roell KR, Reif DM, Motsinger-Reif AA. 2017. An introduction to terminology and methodology of chemical synergy-perspectives from across disciplines. Front Pharmacol 8:158. doi:10.3389/fphar.2017.00158 28473769 PMC5397413

[23] Larkins-Ford J, Degefu YN, Van N, Sokolov A, Aldridge BB. 2022. Design principles to assemble drug combinations for effective tuberculosis therapy using interpretable pairwise drug response measurements. Cell Rep Med 3:100737. doi:10.1016/j.xcrm.2022.100737 36084643 PMC9512659

[24] Katzir I, Cokol M, Aldridge BB, Alon U. 2019. Prediction of ultra-high-order antibiotic combinations based on pairwise interactions. PLoS Comput Biol 15:e1006774. doi:10.1371/journal.pcbi.1006774 30699106 PMC6370231

[25] Walter ND, Born SEM, Robertson GT, Reichlen M, Dide-Agossou C, Ektnitphong VA, Rossmassler K, Ramey ME, Bauman AA, Ozols V, et al.. 2021. Mycobacterium tuberculosis precursor rRNA as a measure of treatment-shortening activity of drugs and regimens. Nat Commun 12:2899. doi:10.1038/s41467-021-22833-6 34006838 PMC8131613

[26] Conradie F, Diacon AH, Ngubane N, Howell P, Everitt D, Crook AM, Mendel CM, Egizi E, Moreira J, Timm J, McHugh TD, Wills GH, Bateson A, Hunt R, Van Niekerk C, Li M, Olugbosi M, Spigelman M, Nix-TB Trial Team. 2020. Treatment of highly drug-resistant pulmonary tuberculosis. N Engl J Med 382:893–902. doi:10.1056/NEJMoa1901814 32130813 PMC6955640

[27] Conradie F, Bagdasaryan TR, Borisov S, Howell P, Mikiashvili L, Ngubane N, Samoilova A, Skornykova S, Tudor E, Variava E, et al.. 2022. Bedaquiline-pretomanid-linezolid regimens for drug-resistant tuberculosis. N Engl J Med 387:810–823. doi:10.1056/NEJMoa2119430 36053506 PMC9490302

[28] Bowness R, Boeree MJ, Aarnoutse R, Dawson R, Diacon A, Mangu C, Heinrich N, Ntinginya NE, Kohlenberg A, Mtafya B, Phillips PPJ, Rachow A, Plemper van Balen G, Gillespie SH. 2015. The relationship between Mycobacterium tuberculosis MGIT time to positivity and cfu in sputum samples demonstrates changing bacterial phenotypes potentially reflecting the impact of chemotherapy on critical sub-populations. J Antimicrob Chemother 70:448–455. doi:10.1093/jac/dku415 25344806

[29] Dooley KE, Hanna D, Mave V, Eisenach K, Savic RM. 2019. Advancing the development of new tuberculosis treatment regimens: the essential role of translational and clinical pharmacology and microbiology. PLoS Med 16:e1002842. doi:10.1371/journal.pmed.1002842 31276490 PMC6611566

[30] Dartois VA, Rubin EJ. 2022. Anti-tuberculosis treatment strategies and drug development: challenges and priorities. Nat Rev Microbiol 20:685–701. doi:10.1038/s41579-022-00731-y 35478222 PMC9045034

[31] Tekin E, Savage VM, Yeh PJ. 2017. Measuring higher-order drug interactions: a review of recent approaches. Curr Opin Syst Biol 4:16–23. doi:10.1016/j.coisb.2017.05.015

[32] Tasneen R, Williams K, Amoabeng O, Minkowski A, Mdluli KE, Upton AM, Nuermberger EL. 2015. Contribution of the nitroimidazoles PA-824 and TBA-354 to the activity of novel regimens in murine models of tuberculosis. Antimicrob Agents Chemother 59:129–135. doi:10.1128/AAC.03822-14 25331697 PMC4291340

[33] De Groote MA, Gruppo V, Woolhiser LK, Orme IM, Gilliland JC, Lenaerts AJ. 2012. Importance of confirming data on the in vivo efficacy of novel antibacterial drug regimens against various strains of Mycobacterium tuberculosis. Antimicrob Agents Chemother 56:731–738. doi:10.1128/AAC.05701-11 22143517 PMC3264252

[34] Bigelow KM, Tasneen R, Chang YS, Dooley KE, Nuermberger EL. 2020. Preserved efficacy and reduced toxicity with intermittent linezolid dosing in combination with bedaquiline and pretomanid in a murine tuberculosis model. Antimicrob Agents Chemother 64:e01178-20. doi:10.1128/AAC.01178-20 32690647 PMC7508620

[35] Bialek W, Cavagna A, Giardina I, Mora T, Silvestri E, Viale M, Walczak AM. 2012. Statistical mechanics for natural flocks of birds. Proc Natl Acad Sci U S A 109:4786–4791. doi:10.1073/pnas.1118633109 22427355 PMC3324025

[36] Beppler C, Tekin E, White C, Mao Z, Miller JH, Damoiseaux R, Savage VM, Yeh PJ. 2017. When more is less: emergent suppressive interactions in three-drug combinations. BMC Microbiol 17:107. doi:10.1186/s12866-017-1017-3 28477626 PMC5420147

[37] Tekin E, White C, Kang TM, Singh N, Cruz-Loya M, Damoiseaux R, Savage VM, Yeh PJ. 2018. Prevalence and patterns of higher-order drug interactions in Escherichia coli. NPJ Syst Biol Appl 4:31. doi:10.1038/s41540-018-0069-9 30181902 PMC6119685

[38] Singh N, Yeh PJ. 2017. Suppressive drug combinations and their potential to combat antibiotic resistance. J Antibiot (Tokyo) 70:1033–1042. doi:10.1038/ja.2017.102 28874848 PMC5659931

[39] Jones A, Saini J, Kriel B, Via LE, Cai Y, Allies D, Hanna D, Hermann D, Loxton AG, Walzl G, Diacon AH, Romero K, Higashiyama R, Liu Y, Berg A. 2022. Sputum lipoarabinomannan (LAM) as a biomarker to determine sputum mycobacterial load: exploratory and model-based analyses of integrated data from four cohorts. BMC Infect Dis 22:327. doi:10.1186/s12879-022-07308-3 35366820 PMC8976459

[40] Honeyborne I, McHugh TD, Phillips PPJ, Bannoo S, Bateson A, Carroll N, Perrin FM, Ronacher K, Wright L, van Helden PD, Walzl G, Gillespie SH. 2011. Molecular bacterial load assay, a culture-free biomarker for rapid and accurate quantification of sputum Mycobacterium tuberculosis bacillary load during treatment. J Clin Microbiol 49:3905–3911. doi:10.1128/JCM.00547-11 21900522 PMC3209113

[41] Diacon AH, Donald PR. 2014. The early bactericidal activity of antituberculosis drugs. Expert Rev Anti Infect Ther 12:223–237. doi:10.1586/14787210.2014.870884 24392698

[42] Boeree MJ, Lange C, Thwaites G, Paton N, de Vrueh R, Barros D, Hoelscher M. 2021. UNITE4TB: a new consortium for clinical drug and regimen development for TB. Int J Tuberc Lung Dis 25:886–889. doi:10.5588/ijtld.21.0515 34686229 PMC8544922

[43] Lenaerts AJ, Gruppo V, Marietta KS, Johnson CM, Driscoll DK, Tompkins NM, Rose JD, Reynolds RC, Orme IM. 2005. Preclinical testing of the nitroimidazopyran PA-824 for activity against Mycobacterium tuberculosis in a series of in vitro and in vivo models. Antimicrob Agents Chemother 49:2294–2301. doi:10.1128/AAC.49.6.2294-2301.2005 15917524 PMC1140539

[44] Gabrielsson J, Weiner D. 2007. Pharmacokinetic and pharmacodynamic data analysis: concepts and applications. Fourth edition. Swedish Pharmaceutical Press, Stockholm, Sweden.

[45] Mould DR, Upton RN. 2012. Basic concepts in population modeling, simulation, and model-based drug development. CPT Pharmacometrics Syst Pharmacol 1:e6. doi:10.1038/psp.2012.4 23835886 PMC3606044

[46] Lyons MA. 2019. Modeling and simulation of pretomanid pharmacodynamics in pulmonary tuberculosis patients. Antimicrob Agents Chemother 63:e00732-19. doi:10.1128/AAC.00732-19 31570404 PMC6879235

[47] Bois FY. 2009. GNU MCSim: bayesian statistical inference for SBML-coded systems biology models. Bioinformatics 25:1453–1454. doi:10.1093/bioinformatics/btp162 19304877

[48] Tendler A, Zimmer A, Mayo A, Alon U. 2019. Noise-precision tradeoff in predicting combinations of mutations and drugs. PLoS Comput Biol 15:e1006956. doi:10.1371/journal.pcbi.1006956 31116755 PMC6548401

