# Supplementary material for: Use of multiple pharmacodynamic measures to deconstruct the Nix-TB regimen in a short-course murine model of tuberculosis
Source: Antimicrob Agents Chemother. 2024 Mar 19;68(5):e01010-23. doi: 10.1128/aac.01010-23 (PMC11064538; doi:10.1128/aac.01010-23)
Supplement: Supplemental material — Untreated group data and model parameters. [file aac.01010-23-s0007.pdf]

SUPPLEMENTARY MATERIAL

Use of Multiple Pharmacodynamic Measures to Deconstruct the Nix-TB Regimen in a Short-course Murine Model of Tuberculosis

M.A. Lyons[1], A. Obregon-Henao[1], M.E. Ramey[1], A.A. Bauman[1], S. Pauly[2], K. Rossmassler[2], J. Reid[2], B. Karger[1], N.D. Walter[2,3,4], and G.T. Robertson[1,3].

[1] Mycobacteria Research Laboratories, Department of Microbiology, Immunology and Pathology, Colorado State University, Fort Collins, Colorado, USA

[2] Division of Pulmonary Sciences and Critical Care Medicine, University of Colorado Anschutz Medical Campus, Aurora, CO, USA

[3] Consortium for Applied Microbial Metrics, Aurora, CO, USA

[4] Rocky Mountain Regional VA Medical Center, Aurora, CO, USA

Figure S1: Untreated group data and model simulation.

Data File S1: MCSim model definition file

Data File S2: MCSim MCMC simulation file

Data File S3: MCSim model simulation file

Data File S4: Observed mouse CFU data

Data File S5: Observed mouse RS ratio data

Data File S6: Observed mouse TTP data

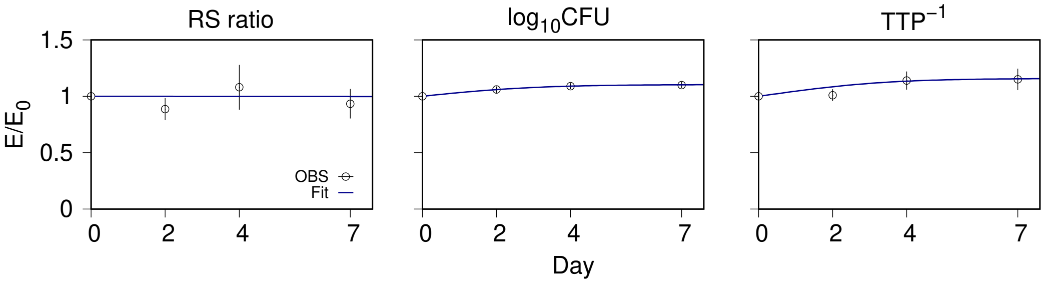

Figure S1: Untreated group data. Fractional effect (E/E_0) for the RS ratio, $\log_{10}$CFU, and reciprocal of the time to positivity (TTP^{-1}) versus treatment day. Model simulations of logistic growth (solid lines) with growth rate constant $\mu = 0.72$/d and carrying capacity $K = 9.34 \times 10^7$ CFU/lung, together with the observed (OBS) group mean (points) and SD (error bars) The baseline (E_0) values (mean [SD], n=7 mice) were 211 (17.8) (ETS1/23S/10^4) for RS ratio, 7.22 (0.136) $\log_{10}$ CFU/lung, and 151 (3.26) h for TTP.
